# Successful Detection of Unrecognized *Rickettsia typhi* in Pregnancy Using Cell-Free Next-Generation Sequencing

**DOI:** 10.1155/2020/6767351

**Published:** 2020-05-26

**Authors:** Irene A. Stafford, Fernando H. Centeno, Mayar Al Mohajer, George Parkerson, Laila Woc-Colburn, Angelica Janice Burgos-Lee, Martha Rac, James Dunn, Kenneth Muldrew

**Affiliations:** ^1^Department of Obstetrics and Gynecology, Baylor College of Medicine, Houston, TX, USA; ^2^Baylor College of Medicine and Texas Children's Hospital, Houston, TX, USA; ^3^Section of Infectious Diseases, Baylor College of Medicine, Houston, TX, USA; ^4^National School of Tropical Medicine, Baylor College of Medicine, Houston, TX, USA; ^5^Department of Pharmacy, Baylor College of Medicine, Houston, TX, USA; ^6^Department of Pathology and Immunology, Baylor College of Medicine, Houston, TX, USA

## Abstract

Flea-borne (murine) typhus is caused by *Rickettsia typhi*. Infection in pregnant women can lead to adverse outcomes when diagnosis and treatment is delayed. We describe how next-generation sequencing (NGS) using the Karius® test was used to rapidly diagnose murine typhus in two pregnant women admitted to a large tertiary care center in Houston, Texas, when all initial testing was nondiagnostic.

## 1. Background

Murine typhus is a Rickettsial disease caused by the bacteria *Rickettsia typhi* (*R*. *typhi*) or possibly *R*. *felis* and can be transmitted by infected fleas harbored by rats, cats, dogs, and other small mammals [1–5]. In the United States, Texas reports the highest numbers of flea-borne typhus cases annually with the majority of cases occurring in the southern coastal region [2–5]. In 2018, there were 738 reported cases in the state of Texas, representing a 2-fold increase compared to 2016 [1]. Clinical presentation often includes fever, headache, rash, nausea, vomiting, and myalgias accompanied by abnormal hematologic indices including anemia (18–75%), thrombocytopenia (19–48%), elevated aminotransferase levels (38–90%), and an elevated erythrocyte sedimentation rate (59–89%) [3–5]. Approximately 14-28% of infected people have evidence of hepatomegaly or splenomegaly, and in severe cases, pulmonary and cardiac compromise can occur [2–5]. Several case reports and series of murine typhus infection in pregnant women have been published with varying reports of adverse perinatal outcomes, ranging from a mild self-limited disease course to a 40% incidence of preterm birth and low birth weight [6–18]. This current report would total the number of published cases reported in pregnancy to 100 and is only the second published case report of murine typhus infection during pregnancy in women living in the southwestern region of the United States [6–18]. Diagnosis in pregnancy is often challenging given the overlap of initial hematologic results with other infectious diseases and critical obstetrical conditions that are more common [3–8]. In addition, initial serologic titers using indirect immunofluorescence assay (IFA) IgM testing are often inconclusive, as only 50% of patients will have serologic evidence of disease one week after onset of infection [5]. Often, the diagnosis is delayed, as most infected people demonstrate seroconversion two weeks after onset of illness; therefore, repeat testing is recommended [3–7, 19]. A single titer of 1 : 128 is considered diagnostic for infection [3–5]. Similar to other published manuscripts describing this infection during pregnancy, we report two cases presenting with a potentially life-threatening disease course, supporting the need for prompt diagnosis and treatment. Cell-free next-generation sequencing using the Karius® test was used to rapidly diagnose *R*. *typhus* in both patients, prompting directed therapy to eradicate infection with doxycycline. The Karius® test, performed in a Clinical Laboratory Improvement Amendments- (CLIA-) licensed, College of American Pathologists- (CAP-) accredited laboratory (Karius Inc., Redwood City, CA), is a test that utilizes next-generation sequencing (NGS) to detect circulating microbial cell-free DNA (mcfDNA) in plasma. Blood plasma from a routine draw is isolated and shipped overnight at ambient temperature to the Karius CLIA/CAP laboratory. Sample-specific controls are added on receipt, and an automated liquid-handling platform performs cfDNA extraction and NGS library preparation [20]. The NGS libraries are multiplexed, inspected for quality, and sequenced. A custom-built analysis pipeline uses a clinical-grade database to identify microbial DNA fragments found in plasma [20]. Pathogens with plasma DNA levels that are significantly higher than real-time background thresholds are listed on the patient report, along with the concentration of the microbial cfDNA in plasma reported as molecules per microliter (MPM). In the largest validation study of this platform, the simulated organism was correctly identified in 121 of 125 simulations for a sensitivity of 97.5%. The positive predictive value (PPV) was 99% (121 of 122), consistent with the expected 95% sensitivity at the level of detection. These findings have been validated with research published in the clinical setting using appropriate comparetors [20–23].

## 2. Case Presentation

### 2.1. Case 1

The patient is a 34-year-old pregnant white female who presented at 31 4/7 weeks gestation to an outside emergency department (ED) with complaints of unrelenting headaches, rash, and a fever of 103.1°F. She was experiencing these symptoms for two days at home. She reported the rash was limited to her trunk and arms, mainly characterized by small red bumps that were nonpruritic. She reported her headache to be mainly frontal and mildly relieved with acetaminophen. She was employed as a veterinarian technician and left work early that day due to her symptoms. She was discharged with an antipyretic and instructed to follow up with her obstetrician. After 24 hours, she represented to the same ED with continued complaints of malaise, fever, and arthralgia. At that visit, her laboratory results demonstrated elevated liver enzymes and thrombocytopenia. The diagnosis of a nonspecific viral syndrome was made, and the patient was discharged to home. On the third day, after the initial presentation to the ED, the patient presented to the obstetrical triage unit with worsening symptoms and a fever of 103.4°F. Results of her blood work at that time revealed elevated liver enzymes, proteinuria, thrombocytopenia, and a significantly decreased white blood count (Table 1). She was transferred to the intensive care unit, and vancomycin, piperacillin/tazobactam, and azithromycin were initiated for broad antimicrobial coverage. Fetal ultrasound revealed an appropriately grown nonanomalous fetus with normal amniotic fluid. Blood and urine cultures along with a respiratory PCR panel for respiratory syncytial virus, adenovirus, influenza A/B, and parainfluenza virus were performed. Serology testing for pathogens including viral hepatitis, syphilis, human immunodeficiency virus, cytomegalovirus, herpes simplex virus, Epstein-Barr virus (EBV), coccidioidomycosis, histoplasmosis, and parvovirus was ordered (Table 1). After 48 hours, all results were negative, including the IFA serologic IgM testing for *Rickettsia typhi*. On her third hospital stay, she began experiencing dyspnea and desaturations to 88-91%. A chest film was ordered and revealed bilateral opacities in then lower lung fields, and emergent computed tomography (CT) demonstrated similar findings with no evidence of thrombus. An echocardiogram revealed normal cardiac function with an ejection fraction > 55%. With impending respiratory failure and continued febrile morbidity, the multidisciplinary team ordered the Karius® test. Twenty-four hours after sample receipt, the test reported the detection of *Rickettsia typhi* at 147 cfDNA MPM with no other pathogens identified. After a thorough discussion with the patient and her family about risks and benefits, oral doxycycline 100 mg twice daily for 7 days was initiated per the Centers for Disease Control and Prevention recommendations for the treatment of murine typhus and all other antibiotics were discontinued [19]. Within 24 hours, the patient demonstrated symptomatic improvement, her oxygen requirement was improved, and labs began to normalize. The patient required physical therapy and aggressive pulmonary care for several days before she was discharged to home to complete the course of doxycycline. Although preliminarily negative, murine typhus was confirmed with IFA serologic IgM and IgG titer values of >1 : 256 and >1 : 128, respectively, three weeks after admission. Follow-up studies demonstrated clearance of *Rickettsia typhi* using these standard serologic testing platforms. She delivered a full-term male infant with a reassuring newborn assessment without any complications.

### 2.2. Case 2

The patient is a 29-year-old white female pregnant at 13 1/7 weeks gestation who presented to an outpatient clinic complaining of a headache and a fever recorded as 102.7°F. She denied sick contacts and lived with her partner and two household pets that were recently treated for fleas. A PCR and throat culture for Group A Streptococcus along with a respiratory PCR panel for influenza A/B, respiratory syncytial virus, adenovirus, and parainfluenza virus were performed, and the patient was instructed to follow up for worsening symptoms when results were reportedly negative. Two days later, she presented to the obstetrical triage unit complaining of worsening symptoms including continued subjective fever, headache, and cough. A urinalysis was performed, and she was discharged to home with an antipyretic and nitrofurantoin for a urinary tract infection. She returned 24 hours later with worsening dyspnea, shortness of breath, and a fever of 102.9°F. Laboratory testing revealed elevated liver enzymes and leukocytosis with a left shift (Table 1). Fetal ultrasound revealed an appropriately grown fetus with normal amniotic fluid. Chest X-ray revealed bibasilar infiltrates, and a chest CT revealed similar results with no evidence of thrombus. A right upper quadrant scan revealed hepatomegaly. She was started on broad-spectrum antibiotics including vancomycin, meropenem, and azithromycin. An extensive laboratory evaluation including a repeat respiratory panel; blood, urine, and stool cultures; and serology testing for viral hepatitis, syphilis, human immunodeficiency virus, cytomegalovirus, herpes simplex virus, EBV, coccidioidomycosis, histoplasmosis, and *Rickettsia typhi* was performed, and results were all initially negative except for positive EBV IgG serology. These results were consistent with past infection given the negative viral capsule antigen (VCA) IgM serology result, clinical exam, and patient history. The patient was transferred to the intensive care unit for a higher level of care. With continued symptoms and febrile morbidity, the multidisciplinary team involved in her case ordered the Karius® test.

Twenty-four hours after sample receipt, the test detected *Rickettsia typhi* at 6 cfDNA MPM in the raw data which was under the commercial threshold but available through clinical consultation. No reads of *R*. *typhi* are found in the plasma by Karius® testing in a cohort of 684 self-reported healthy controls. Despite the low level of *R*. *typhi*, the diagnosis of murine typhus was determined by the team of providers caring for the patient based on the clinical presentation, the unreliability of early diagnostic tests for *R*. *typhi*, and the epidemiologic setting of high flea exposure in a potentially infested apartment complex. The Karius® test detected EBV consistent with serologic results indicative of past disease, and no other organisms were detected. With informed consent, 100 mg oral doxycycline twice daily was initiated for treatment of murine typhus and all other antibiotics were discontinued. Within 18 hours, the patient demonstrated symptomatic improvement, and after 24 hours, her laboratory results began to normalize. The patient was discharged to home 48 hours later to complete a 10-day course of doxycycline per CDC guidelines, with markedly improved chest X-ray findings and laboratory results. On day 10 after admission, IFA serologic testing confirmed the diagnosis of *R*. *typhi* and the patient was followed with serial serologic testing until clearance of infection was noted. Her pregnancy has otherwise been uncomplicated, and she remains undelivered at the time this report was written.

## 3. Discussion

Murine typhus is an uncommon bacterial infection caused by *Rickettsia typhi*, which is traditionally carried through infected fleas that transmit infection from rodents to humans [3–7]. Recently, endemic foci of murine typhus in Texas and California have been reported and are largely explained by exposure of humans to infected fleas from opossums and cats that serve as a reservoir, explaining infection in these two cases where exposure to flea-infested cats and small animals was present [1–3]. Rates of murine typhus in Texas have increased yearly with a record high of 738 cases reported in 2018 [1]. Symptoms are often nonspecific, and the classic triad of fever, rash, and headache are present only approximately 30% of the time when a patient presents for care [4, 5]. Other signs and symptoms include malaise, chills, conjunctivitis, and splenomegaly in up to 67% of patients. Laboratory results demonstrate elevated liver enzymes, thrombocytopenia, leukopenia, elevated lactate dehydrogenase, and hypoalbuminemia [4, 5]. Significant overlap exists in the symptomatology of this infection with other Rickettsial diseases, viral syndromes, and even critical obstetrical complications such as preeclampsia, where transaminitis and thrombocytopenia are common [4–18]. Approximately 25% of infected people may have more severe manifestations of disease including respiratory or renal failure, aseptic meningitis, and shock, requiring aggressive management in the intensive care unit setting [4, 5].

Adverse perinatal outcomes have been described in pregnant women infected with *Rickettsia typhi* including increased rates of miscarriage and poor neonatal outcomes with prolonged febrile morbidity and disease; however, congenital infection and related fetal risks remain poorly defined [4–18]. In a large series of over 97 gravid patients infected with *Rickettsia typhi*, a poor neonatal outcome was recorded in over 40% of deliveries and 2% of cases were associated with maternal death [4–18].

The standard for diagnosis of *Rickettsia typhi* is the detection of IgM antibodies by indirect immunofluorescence assay (IFA); however, IFA IgM is only positive in half of infected patients during the first week of infection [4, 5, 19]. Polymerase chain reaction (PCR) technology or bacterial isolation from a clinical specimen can also be used for laboratory diagnosis; however, these tests lack clinical sensitivity and are not available in all settings [4–8, 19]. Technologies using cell-free next-generation sequencing have provided novel methods to rapidly identify infectious agents including the Rickettsial species [20–23]. The utility of this technique has been demonstrated in the detection of pathogenic bacteria, fungi, and viruses in clinical samples, when routine diagnostic methods were negative, ultimately facilitating targeted treatment and recovery [20–23]. In a pilot study involving pediatric immunocompromised patients, next-generation sequencing from microbial cell-free DNA isolated from human plasma detected 75% of bloodstream infections days prior to the onset of attributable symptoms [20–23]. This testing modality also detected the causative pathogen in 87% of pediatric patients hospitalized with community-acquired pneumonia compared to standard culture and PCR technology, facilitating a change in antibiotic regimens in 47% of cases [20–23].

The Karius® platform for next-generation sequencing for the detection and identification of pathogens relies on the detection of microbial cell-free DNA detected in human plasma. The sensitivity of detection of approximately 40 molecules per microliter allows for results of near certainty, especially when the illness is characterized by nonspecific findings and a wide differential diagnosis with inconclusive preliminary results [20–23]. An additional advantage of this testing platform is the speed in which results are available for the testing provider. When considering the progression of disease that may be associated with *Rickettsia typhi* infection in pregnancy, rapid results can facilitate expeditious treatment with appropriate antimicrobial therapy as described in this case, where both patients initially tested negative for *R*. *typhi*-specific IFA IgM. Although the Karius® test was negative in the second case, the low observation of 6 molecules per microliter of *R*. *typhus* cfDNA may have represented early infection which ultimately was confirmed with subsequent serologic confirmation. Of note, positive EBV detected by the Karius® test was also confirmed with serologic evaluation however represented prior disease. Quantities of mcfDNA between microorganisms should not be compared. Genomes sizes are different—especially between Kingdoms—and the MPM may represent different pathogen loads depending on the specific organism. In addition, the differential burden, natural history, and pretreatment or control of each infecting agent may be quite variable. Reliance on interpretation of confirmatory serologic analysis and sound clinical judgment cannot be overstated, especially if more than one pathogen is detected. Although not currently approved by the FDA, the test is provided by Karius Inc. which is a Clinical Laboratory Improvement Amendments- (CLIA-) licensed, College of American Pathologists- (CAP-) accredited laboratory (Karius Inc., Redwood City, CA).

Randomized trials to assess the effectiveness of various antibiotic regimens in pregnant patients with murine typhus are limited, but clinical experience and strong evidence support the use of doxycycline as the first-line antibiotic treatment, even in the gravid patient [5, 24–26]. Azithromycin has demonstrated in vitro benefit but remains less effective in most clinical cases, as demonstrated in these 2 cases where both patients did not respond to azithromycin [24–26]. The use of doxycycline is associated with a decrease in the average length of febrile illness from approximately 2 weeks to less than 4 days which prevents infection-related sequelae and worsening of disease in both mother and newborn [24–26]. The recent literature reviewed on use of doxycycline in pregnant women with Rickettsial infections demonstrates superior efficacy and relative safety [24–28]. A systematic review of the available literature on doxycycline use in pregnant women revealed a safety profile of doxycycline that differed significantly from that of tetracycline, with no correlation between the use of doxycycline and teratogenic effects [25, 26]. Although tetracyclines have been associated with higher rates of neural tube defects, cleft palate, and other major congenital abnormalities, there is no evidence in the literature of any human teratogenicity following the use of doxycycline during pregnancy, including tooth discoloration for exposed children [26]. During the time period of bioterrorism threat in the United States, doxycycline was FDA-approved for use in pregnant women following exposure to biothreat agents, including *Bacillus anthracis*, *Yersinia pestis*, and *Francisella tularensis* [29].

This support was echoed by the American College of Obstetrics and Gynecology in a practice bulletin published in 2009 [27]. In a large retrospective analysis of over 30,000 deliveries in the United States, multivariable analysis provided no evidence of greater risk for malformations in infants with fetal exposure to doxycycline than unexposed infants during the first 4 months of pregnancy (RR: 0.85) or anytime during pregnancy (RR: 0.84) [25, 26]. In 2009, the US FDA stated that while the risks of doxycycline were minimal, the risk of associated tooth staining could not be disregarded and required more adverse event data [28, 29]. As a result, the drug remains a pregnancy risk category class D, despite the recommendation that doxycycline is the first-line therapy for pregnant women with Rocky Mountain Spotted Fever [28, 29].

Emerging data on doxycycline responsive illnesses, such as *Mycoplasma*, *Chlamydia*, and *Rickettsial* species such as murine typhus and their associated morbidity in pregnancy, are strong arguments to revisit and reconsider the indications for the use of this inexpensive and widely available antibiotic. If *Rickettsia typhi* infection presents during pregnancy, a robust risk benefit discussion surrounding therapy with doxycycline should occur, as a superior available alternative does not exist for the gravid patient at risk for decompensation [5]. This would include a discussion describing the paucity of data surrounding teratogenicity of doxycycline and the currently unknown risk of in utero exposure on permanent dental staining of exposed offspring versus the risks of murine typhus infection in pregnancy, which is associated with a near 40% risk of decompensation and potential adverse perinatal outcomes. Doxycycline has the potential to make a significant impact on perinatal morbidity and mortality locally and worldwide.

## 4. Conclusion

This report describes the clinical course of two pregnant women infected with murine typhus during pregnancy in a large tertiary care hospital in Houston, Texas. The utilization of the Karius® test, a cell-free next-generation sequencing assay, resulted in a timely and accurate diagnosis when conventional testing platforms were nondiagnostic. Similar to prior published research regarding therapeutic options for *Rickettsia typhi*, the use of doxycycline proved highly efficacious.

## Figures and Tables

**Table 1:** Patients with *Rickettsiatyphi* detection by NGS of plasma-based mcfDNA during pregnancy.

Parameter	Case 1	Case 2
Age (years)	34	29
Pregnancy details	G3P2, 31 3/7 wks GA	G1P0, 13 1/7 wks GA
Associated conditions		
Initial suspected diagnosis	Viral syndrome	Viral syndrome, UTI
Fever	103.1 F→103.4 F	102.7 F→102.9 F
Septic shock	Yes	Yes
ICU admission	Hospital day 3	Hospital day 3
Headache	Yes	Yes
Confusion	No	No
Rash	Yes	No
Myalgia/arthralgia	Yes	No
Malaise	Yes	Yes
Respiratory symptoms	Dyspnea, hypoxia	Dyspnea, cough
GI symptoms	Yes	No
Hepatomegaly/splenomegaly	Imaging not performed	Hepatomegaly
Myocarditis	No	No
Laboratory analysis		
Hgb (Ref: 9.6-12.4 g/dL)	9	12
WBC (Ref: 6.00-13.32 cells/mm^3^)	2.5	11.6
%PMNs (Ref: 10.9-76.0%)	33.1	61.4
%Bands	43.7	11.8
Platelet count (10^3^/*μ*L) (Ref: 244-580 10^3^/*μ*L)	91	354
Na (Ref: 133-142 mEq/L)	136	135
BUN (Ref: 2–23 mg/dL)	10	13
Cr (Ref: 0.15-0.4 mg/dL)	0.51	0.62
AST (Ref: 20-60 U/L)	651	95
ALT (Ref: 12-45 U/L)	429	146
Alk Phos (Ref: 60-330 U/L)	79	62
Lactate dehydrogenase (313-618 U/L)	860	774
Hematuria	Yes	No
Proteinuria	Yes	No
Lumbar puncture	Not performed	Not performed
Imaging	U/S: normal fetus CXR: bibasilar opacities	U/S: normal fetus CXR: bibasilar infiltrates Abd U/S: hepatomegaly
Karius® test result *Rickettsia typhi* cfDNA MPM (RR 0 MPM) EBV cfDNA MPM (RR 1.38 MPM)	147	6∗ 242
*Rickettsia typhi* indirect immunofluorescence assay (IFA) serology IgG and IgM	Negative day 3 IgM (<1 : 64) Positive day 28 IgM 1 : 256 IgG 1 : 128	Negative day 3 IgM (<1 : 64) Positive day 10 IgM 1 : 128 IgG 1 : 1024
Blood, throat, stool, urine Cxs	Negative	Negative
Rapid plasma reagin	Negative	Negative
Hepatitis panel serology IgG and IgM	Negative	Negative
Human immunodeficiency virus 1/2 Ag/Ab 4^th^ gen with reflex	Negative	Negative
Herpes simplex virus PCR direct assay	Negative	Negative
Epstein-Barr virus antibody panel IgM and IgG to EBV VCA	Negative	Positive—EBV VCA IgM < 10 VCA IgG 1 : 544 EBNA IgG > 600
Cytomegalovirus IgG and IgM	Negative	Negative
Parvovirus IgM	Negative	Negative
Toxoplasma gondii IgG and IgM	Negative	Negative
Coccidioides IgM to DTP antigen	Negative	Negative
QuantiFERON®-TB	Negative	Negative
Histoplasma immunodiffusion of ant M/H	Negative	Negative
Brucella Ab (total) by agglutination	Negative	Negative
Legionella pneumophila urine antigen	Negative	Negative
Coxiella burnetii (Q fever) IgG	Negative	Negative
Empiric antibiotic coverage	Vancomycin, piperacillin/tazobactam, azithromycin	Vancomycin, meropenem, azithromycin
Hospital Day KT ordered	3	2
KT time to diagnosis	1 day from sample receipt 3 days from collection	1 day from sample receipt 2 days from collection
Impact of KT on therapy	Narrow to doxycycline	Narrow to doxycycline
Hospital day of doxycycline initiation	4	3
Outcome	Full recovery, delivery of a full-term male infant	Full recovery, currently 32 wks GA

wks = weeks; RP = rapid respiratory panel; CSF = cerebrospinal fluid; Cx = culture. MPM refers to molecules of microbial cell-free DNA/microliter (of a particular pathogen). RR refers to the reference range in MPM of a particular pathogen which is the 97.5%ile of the Karius® test result for that particular pathogen across a cohort of 609 asymptomatic healthy adults. ∗ indicates detection under the Karius® test commercial threshold.
